# Unveiling the mechanism of deformation-induced supersaturation

**DOI:** 10.1038/s41598-024-66164-0

**Published:** 2024-07-02

**Authors:** Karoline S. Kormout, Lorenz Romaner, Daniel Scheiber, Stefan Zeiler, Reinhard Pippan, Andrea Bachmaier

**Affiliations:** 1 Erich Schmid Institute of Materials Science, Austrian Academy of Sciences, Leoben, Austria; 2 Department of Materials Science, Montanuniversität, Leoben, Austria; 3 Materials Center, Leoben Forschung GmbH, Leoben, Austria; 4 Present Address: Department of Materials Science, Montanuniversität Leoben, Leoben, Austria

**Keywords:** Synthesis and processing, Theory and computation

## Abstract

A Cu-6at%Ag cast alloy was deformed by means of high-pressure torsion to different applied strain levels until a steady-state regime is reached. The continuous structural refinement is attended by the successive dissolution of the Ag precipitates in the Cu matrix. The results show that the Ag regions need to fall below a phase size of ~ 5 nm to fully dissolve. Atomistic calculations indicate that the final dissolution can be explained based on the enthalpy difference between the solid solution and layered systems which are in between the coherent and semi-coherent structure. These findings are supported by detailed microstructural investigations.

## Introduction

Severe plastic deformation (SPD) not only allows to generate ultrafine-grained or nanocrystalline materials, but also enables the formation of metastable, so-called non-equilibrium phases. A prominent example is the deformation-induced intermixing of immiscible elements far beyond thermodynamic equilibrium. Numerous binary systems have been investigated by applying different SPD techniques, such as Cu–Cr, Cu–W and Cu–Fe^1–3^. However, the fundamentals of deformation-induced supersaturation of usually immiscible elements are still a controversial topic in the material science community^4,5^. One open issue is the underlying atomic level intermixing process. Most studies coincide that dislocations play a key role in transferring atoms from one phase to the other. Two main models were proposed, on the one hand the dislocation shuffling model by Raabe et al.^6^, and on the other hand the kinetic roughening model by Bellon and Averback^7^. Both concepts postulate dislocation glide across phase boundaries, a process that requires that certain criteria are fulfilled^8^. The former one considers a Gibbs–Thomson effect by means of high interfacial energies as the final step of dissolution, consequently the phase size needs to fall below a critical level. The latter one assumes an equilibrium between mechanically driven intermixing and decomposition by thermodynamic driving forces, implying that a critical temperature has to be complied. In a previous study on the effect of SPD processing temperature a clear trend from homogenous single-phase supersaturated alloys at low temperatures to phase-separated nanocomposites at elevated temperatures was revealed^9^. In general, during SPD mechanical processes compete with recovery processes, leading to unique steady-state microstructures controlled by complex interactions of material-specific deformation behaviors and phase formations.

However, despite a large number of investigations, no clear experimental evidence for one of the proposed mixing mechanisms could be obtained so far. The motivation for the present study is therefore to gain knowledge about the intermixing process on the atomic level by a comprehensive study on a Cu-6at%Ag cast alloy using transmission electron microscopy (TEM) and synchrotron X-ray diffraction (XRD) experiments complemented with density functional theory (DFT) calculations.

## Results

**Figure 1:**
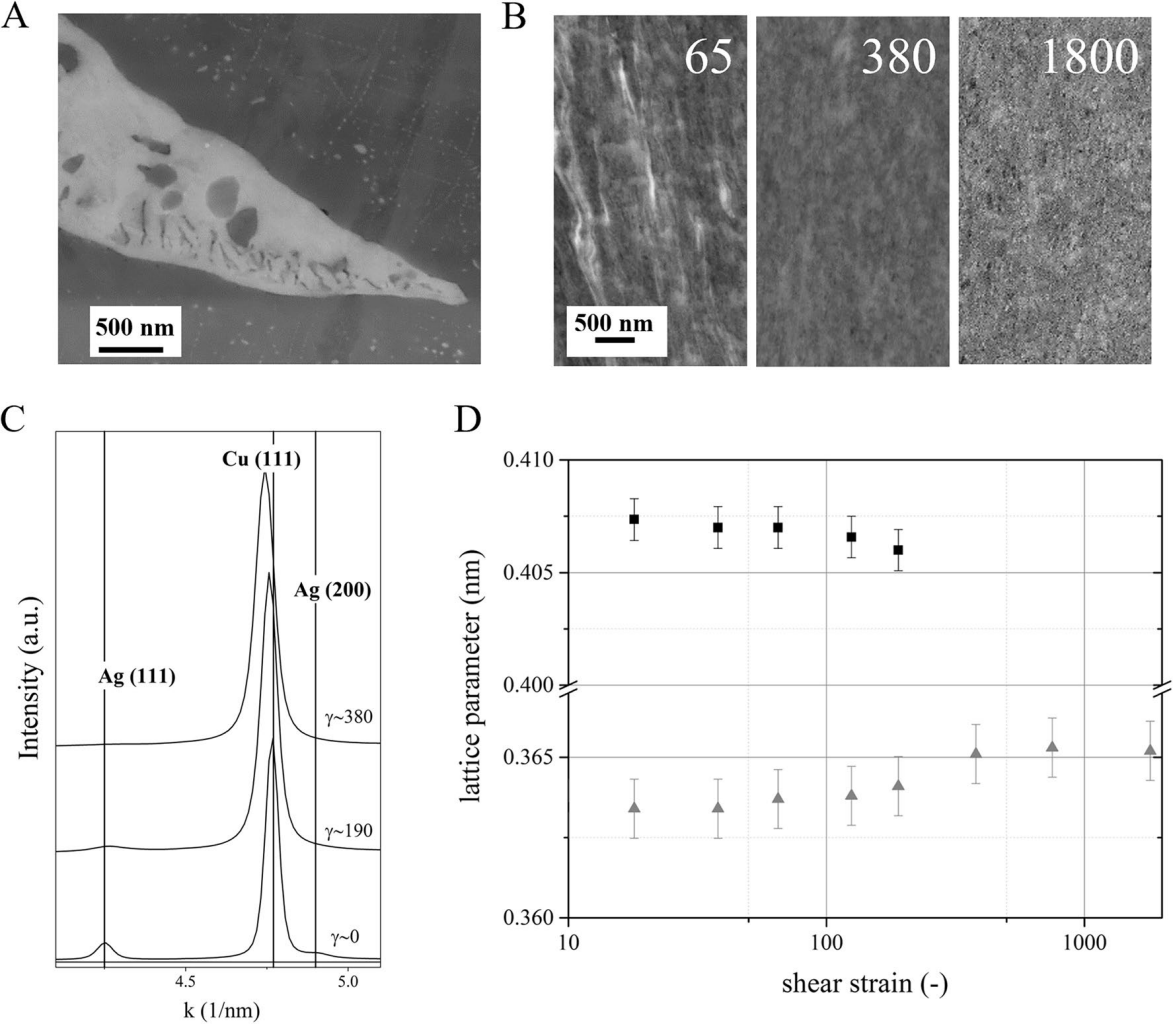
Microstructures of the (**A**) initial cast material and the (**B**) alloy at an applied strain of γ ~ 65, 380 and 1800. The magnification is the same for all three images. (**C**) Synchrotron XRD profiles recorded for different applied strains, γ ~ 0, γ ~ 190 and γ ~ 380 reveal a peak shift for the Cu phase and vanishing Ag peaks. The initial positions of the (111) peaks of Cu and Ag and the (200) peak of Ag are indicated. Please note that the intensities of the profiles were adjusted by a multiplication factor for better visibility. (**D**) Lattice parameter changes of Ag and Cu as a function of applied strain. The error was determined by the pixel size of the used detector.

The initial cast structure of the Cu-6at%Ag alloy (Fig. 1A) exhibits a bimodal size distribution of the Ag precipitates with smaller precipitates around 50 nm and larger ones having diameters around several µm. The continuous structural refinement during HPT deformation leads to a homogenous nanocrystalline alloy (Fig. 1B). The gradual dissolution of the Ag phase in the Cu matrix is evidenced by synchrotron XRD measurements recorded at different strain levels (Fig. 1C). The initial peak position of Cu shifts with increasing applied strain towards smaller k values (thus larger d-spacings) and simultaneously, the Ag peaks diminish until at γ ~ 380 only a single-phase Cu alloy supersaturated with Ag is obtained. The dissolution process is well reflected in the lattice parameter changes obtained by XRD analyses (Fig. 1D). Both Ag and Cu lattice parameters shift away from the initial values indicating a mutual dissolution. At strains higher than γ ~ 380 peaks from the Ag phase are not observed anymore and the Cu lattice parameter remains at a constant level.

**Figure 2:**
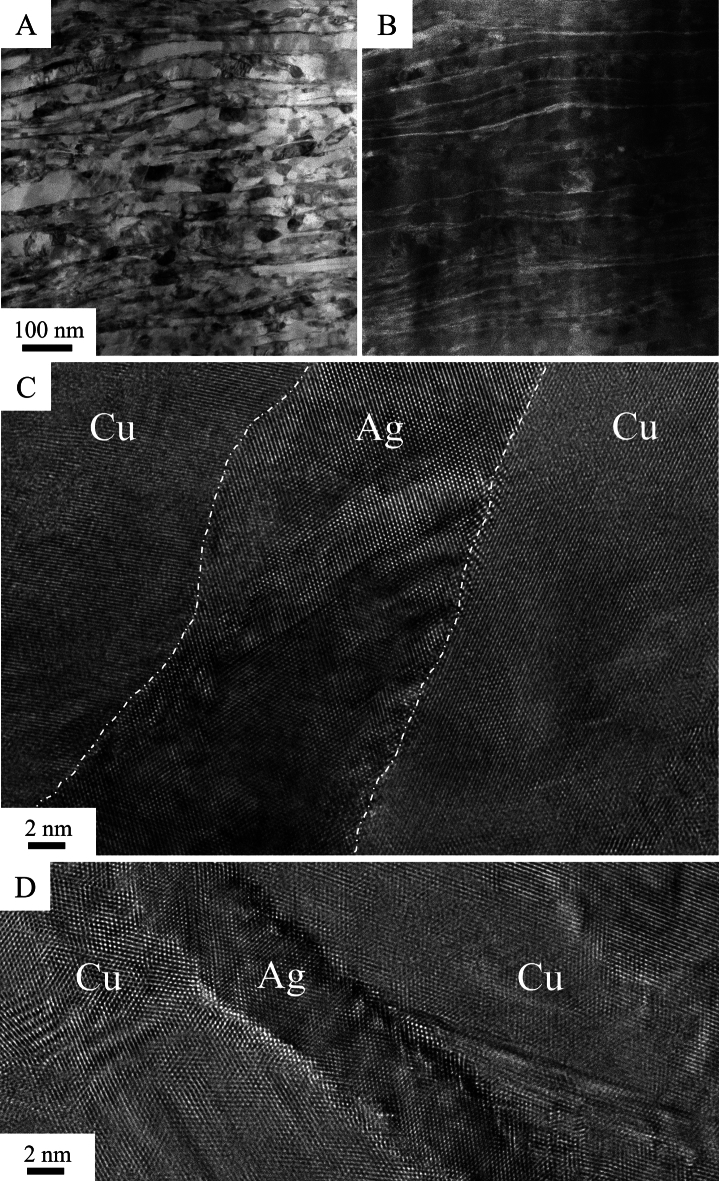
(**A**) STEM BF, (**B**) STEM HAADF, (**C**) and (**D**) HRTEM micrographs of the lamellar Cu-Ag structure at an applied strain of γ ~ 190.

Figure 2 presents typical STEM BF and HAADF micrographs of the microstructural state at an applied strain of γ ~ 190, when the dissolution of Ag has progressed, but is not completed yet. A lamellar configuration can be observed, with thin Ag lamellae embedded in between larger Cu lamellae. In HAADF images Ag regions appear brighter than Cu regions. Typical HRTEM images of Ag lamellae with different thicknesses are shown in Fig. 2C,D. Thin sample regions were chosen to avoid overlapping of Cu and Ag regions, however, because phase boundaries between the lamellae are not oriented perfectly perpendicular to the electron beam some Moiré fringes are visible. Nevertheless, from the HRTEM images it is evident that the phase boundaries remain well-defined and no disordered transition zone between Cu and Ag lamellae is apparent, though a waviness of the boundaries can be noticed. Overall, no lamellae thinner than the ones shown were detected.

**Figure 3:**
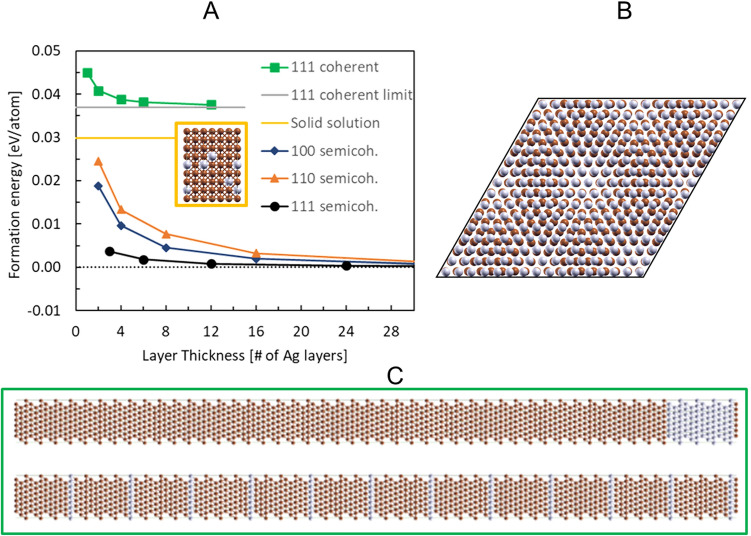
(**A**) Dependence of the formation energy of coherent and semicoherent layered structures as a function of Ag layer thickness. Also shown are the formation energy of the solid solution and the coherent limit corresponding to a hypothetical infinitely large coherent cell with one interface. The inset is a representation of the model used for the solid solution. (**B**) illustration the interface of the (111) incoherent cell (**C**) side view onto two coherent layered structures with 1 and 12 Ag layer thickness.

The results obtained from DFT calculations are compiled in Fig. 3. Layered structures of Cu-8.3at% Ag with varying Ag lamella thickness, from one monolayer Ag to 12 atomic layers of Ag (see Fig. 3C), were calculated and compared to a solid solution which was approximated as a special quasi-random structure (SQS) (Inset in Fig. 3A). The created (111) interfaces are coherent. The formation energy of an atomistic structure in a specific unit cell is calculated as

1 $$E_{f} = E_{cell} - n {\epsilon }_{Cu} - n_{Ag} {\epsilon }_{Ag}$$

where ${E}_{Cell}$ corresponds to the total energy of the cell, ${n}_{Cu}$ and ${n}_{Ag}$ are the amount of Cu and Ag atoms in the cell and ${\epsilon }_{Cu},$ ${\epsilon }_{Ag}$ are the atomic reference energies of the Cu and Ag atoms. They are obtained from a bulk calculation of fcc Cu and Ag in their respective equilibrium lattice constant. The formation energy of the layered structures is plotted as a function of layer thickness in Fig. 3A (green line with rectangles). It is evident that all layered configurations are energetically unstable compared to the solid solution, with the monolayer as the most unfavorable state. In the limit of infinite layer thickness, the formation energy converges to 0.037 eV which is well above the solid solution. Therefore, the solid solution is always energetically favorable compared to the layered structures. From the energy increase an interface energy of 0.12 J/m^2^ can be derived.

Figure 3 also shows the corresponding results for the semi coherent (111) interfaces, which were calculated with an EAM potential. The corresponding supercells contain about 250,000 atoms. Due to the released coherency strains, the formation energies are strongly reduced. In the limit of infinite layer thickness, they converge to zero. For smaller layer thicknesses the formation energy increases but remains still well below the solid solution even at the smallest investigated thickness. A comparable result is obtained for (110) and (100) interfaces, where the increase is more pronounced since the corresponding interface energies are with 0.51 J/m^2^ and 0.43 J/m^2^ higher compared to the 0.14 J/m^2^ of the (111) interface in agreement with previous EAM work^10^. Summarizing, incoherent interfaces are the energetically most favorable state of the system even for very thin Ag slabs consisting of just three atomic layers. This is in contrast to the experimental findings which report a minimal size of the layer of around a few nm suggesting an energetic transition from layered structures to the solid solution at such layer sizes.

## Discussion

In a previous study on a Cu-6at%Ag composite obtained through a powder route a deformation and supersaturation behavior akin to the one observed in the cast material was observed^11^. The major difference between powder and cast composites is the initial microstructure, in particular the size of the Ag regions. The used spherical Ag powder particles have a mean diameter of around 20–50 µm and due the high deformability a lamellar arrangement is already observed after powder compaction with a mean Ag lamella thickness of around 5 µm. Subsequent HPT deformation results in a lamella thickness reduction of both Cu and Ag. Assuming ideal co-deformation, according to^12^ the lamella thickness d at a certain applied strain γ can be calculated with

2 $$d = \frac{{d_{0} }}{\gamma },$$

with d_0_ as initial lamella thickness. Although a deviation from ideal co-deformation could prevail, meaning that thicker lamellae tend to refine faster than thinner ones^11^, nevertheless from this estimation a phase spacing d_s_ for dissolution can be derived. A strain of γ ~ 380 is required for complete intermixing in the Cu-6at%Ag cast alloy, which yields to d_s_ ~ 5 nm, while for the Cu-6at%Ag powder alloy a d_s_ ~ 6 nm is obtained. These values correspond well to the largest lamella sizes observed in TEM imaging (Fig. 2). It appears that the lamella refinement does not continue until Ag reaches a thickness of few atomic layers, instead they seem to dissolve if falling below a critical thickness, which explains why such thin lamellae are not observed experimentally. This assumption is further supported by the results of the DFT calculations. Layers of Ag coherent to the Cu matrix are energetically unfavorable compared to a random solid solution (see Fig. 3) and a clear trend towards instability with decreasing layer thickness is evident.

It is interesting to note that a mutual intermixing of both phases takes place, as revealed from lattice parameter changes, thus also Cu is dissolved into Ag. The dissolution of Cu in Ag could either takes place inside larger Ag precipitates containing small Cu precipitates, as shown in Fig. 1a, or is occurring in general at all Cu/Ag phase boundaries existent. However, a mutual intermixing of Cu and Ag was also observed in the powder composites [not published] and in reference^13^, where Ag regions are well-defined. Therefore, the atomic level intermixing process seems to initiate at phase boundaries, most likely realized by dislocations transferring atoms from one phase to the other. However, when a certain phase dimension is reached, the present results indicate that the last step for intermixing is assisted by dissolution due to high interfacial energies.

Atomistic calculations reveal that surrounding Ag atoms by Cu is energetically unfavorable. This can be seen via fitting a quasi-chemical model to the formation energy of the SQS or to the coherent interface energies. Every Cu–Ag bond is associated with an energy increase of about 0.015 eV compared to the reference Cu–Cu or Ag–Ag bonds. From such bond-considerations alone the solid solution should have the highest energy since it has the highest amount of Cu–Ag bonds per atom. However, this is not the case because of strain energy. The layered coherent structures are strongly expanded or compressed in the 111 planes which adds a strain energy of about 0.037 eV per atom. They are therefore energetically higher in energy compared to the solid solution since strain energy is larger than the bond energy effects.

The energetically most favorable structures would be the semi-coherent structures since they have only slightly higher interface energies compared to the coherent interface but completely removed strain energy. From these findings it would be expected that layers should not transform to solid solution but remain separated even down to monolayer size. The reason why the transition is observed in experiment may have the following reason: A perfectly flat and regular semi-coherent structure as assumed in the calculations is not formed during the mechanical mixing process. Rather the interfaces are wavy and intermediate between the coherent and semi-coherent structures (c.f. Fig. 2C,D). The resulting formation energies in Fig. 3 would be in-between the coherent and semi-coherent limiting cases and intersect the line of the solid solution. This would lead to a transition from layered structures to the solid solution at the intersection point lamellae. Further factors to be taken into account may include different defects densities in the layered structures compared to the solid solution or intermixing effects.

## Conclusion

In summary, during SPD of a Cu-6at%Ag cast alloy a continuous co-deformation results in the supersaturation of Cu and Ag despite a miscibility gap in thermodynamic equilibrium. The experimental results indicate that a phase size of ~ 5 nm for dissolution exists. Although DFT calculations indicate that incoherent interfaces are the energetically most favorable state, the random solid solution may be favored over the layered configuration due the formation of an intermediate state between the coherent and semi-coherent structures. The present findings could give new insights into the atomic mixing behavior of other material systems, such as Cu–W or Cu–Cr, in which mutual solubilities are observed despite very high mixing enthalpies and different crystal structures, impeding dislocation glide across phase boundaries.

## Materials and methods

A Cu-6at%Ag cast alloy (Goodfellow) was deformed by high-pressure torsion (HPT) in quasi-constrained condition^14^ at room temperature. The disk-shaped samples (8 mm in diameter and ~ 0.6 mm in thickness) were subjected to 5 and 10 rotations with a rotation speed of 0.2 rotations per minute, corresponding to applied strains of γ ~ 190 and 380 at a radius of 3 mm. The applied strains can be calculated with

3 $$\gamma = \frac{2\pi rn}{t},$$

with radius r, thickness t of the sample and n being the number of HPT rotations. Microstructural investigations were conducted on an image-side CS-corrected JEOL JEM2100F transmission electron microscope (TEM) obtaining high-resolution TEM (HRTEM) images and scanning TEM (STEM) images in bright-field (BF) and high-angle annular dark-field (HAADF) mode. Synchrotron X-ray diffraction (XRD) experiments were performed at the high energy materials science beamline P07 (operated at the Helmholtz-Zentrum Geesthacht) of the PETRA III synchrotron facility at DESY in Hamburg. The monochromator consists of two bent Si(111) Laue crystals on Rowland geometry. The energy is tunable exactly (33–200 keV**)** with this double crystal monochromator in horizontal scattering geometry. The diffraction measurements were conducted using photon energies of 111 keV and a 2D Perkin-Elmer XRD 1621 detector. A primary slit of 0.5 × 0.5 mm^2^ and secondary slit of 0.7 × 0.7 mm^2^ were used to focus the beam and improve the spatial accuracy of the measurement. The measured transmission diffraction patterns were analyzed with a customized plug-in for Digital Micrograph software^15^. The diffraction patterns of either LaB_6_ or CeO_2_ (NIST references 660b and 674b, respectively) were used as references.

DFT calculations were carried out with the Vienna ab initio simulation package (VASP)^16,17^ using projector augmented wave functions (PAW) and the Perdew–Burke–Ernzerhof exchange–correlation (xc) functional^18^. Cu and Ag were treated with d electrons in the valence and s semi-core electrons. The default energy cut-off supplied with the PAW potentials were used. The convergence criterion for ionic relaxation was 0.01 eV Å^−1^. The k-point mesh was 12 × 12 × 1 for the cells containing the coherent interfaces and 6 × 6 × 4 for the solid solution supercell. All atoms contain 144 atoms. Calculations with the EAM potential^19^ where carried out with LAMMPS^20^.

## Data Availability

The datasets used and/or analyzed during the current study are available from the corresponding author on reasonable request.
